# Effects of subtotal pancreatectomy and long‐term glucose and lipid overload on insulin secretion and glucose homeostasis in minipigs

**DOI:** 10.1002/edm2.425

**Published:** 2023-05-05

**Authors:** Rébecca Goutchtat, Audrey Quenon, Manon Clarisse, Nathalie Delalleau, Anaïs Coddeville, Mathilde Gobert, Valéry Gmyr, Julie Kerr‐Conte, François Pattou, Thomas Hubert

**Affiliations:** ^1^ Univ. Lille, Inserm, CHU Lille, Institut Pasteur Lille, UFR3S, U1190 – Egid Lille France; ^2^ Univ. Lille, CHU Lille, UFR3S, Département Hospitalo‐Universitaire de Recherche et d'Enseignement (Dhure) Lille France; ^3^ GENFIT Loos France

**Keywords:** energetic overload, hyperglycemia, minipig model, pancreatectomy, type 2 diabetes

## Abstract

**Introduction:**

Nowadays, there are no strong diabetic pig models, yet they are required for various types of diabetes research. Using cutting‐edge techniques, we attempted to develop a type 2 diabetic minipig model in this study by combining a partial pancreatectomy (Px) with an energetic overload administered either orally or parenterally.

**Methods:**

Different groups of minipigs, including Göttingen‐like (GL, *n* = 17) and Ossabaw (O, *n* = 4), were developed. Prior to and following each intervention, metabolic assessments were conducted. First, the metabolic responses of the Göttingen‐like (*n* = 3) and Ossabaw (*n* = 4) strains to a 2‐month High‐Fat, High‐Sucrose diet (HFHSD) were compared. Then, other groups of GL minipigs were established: with a single Px (*n* = 10), a Px combined with a 2‐month HFHSD (*n* = 6), and long‐term intraportal glucose and lipid infusions that were either preceded by a Px (*n* = 4) or not (*n* = 4).

**Results:**

After the 2‐month HFHSD, there was no discernible change between the GL and O minipigs. The pancreatectomized group in GL minipigs showed a significantly lower Acute Insulin Response (AIR) (18.3 ± 10.0 IU/mL after Px vs. 34.9 ± 13.7 IU/mL before, *p* < .0005). In both long‐term intraportal infusion groups, an increase in the Insulinogenic (IGI) and Hepatic Insulin Resistance Indexes (HIRI) was found with a decrease in the AIR, especially in the pancreatectomized group (IGI: 4.2 ± 1.9 after vs. 1.5 ± 0.8 before, *p* < .05; HIRI (×10^−5^): 12.6 ± 7.9 after vs. 3.8 ± 4.3 before, *p* < .05; AIR: 24.4 ± 13.7 µIU/mL after vs. 43.9 ± 14.5 µIU/mL before, *p* < .005). Regardless of the group, there was no fasting hyperglycemia.

**Conclusions:**

In this study, we used pancreatectomy followed by long‐term intraportal glucose and lipid infusions to develop an original minipig model with metabolic syndrome and early signs of glucose intolerance. We reaffirm the pig's usefulness as a preclinical model for the metabolic syndrome but without the fasting hyperglycemia that characterizes diabetes mellitus.

## Graphical abstract

In this work, we tried to elaborate a type 2 diabetic minipig model corresponding to the World Health Organization definition by performing a subtotal pancreatectomy and long‐term intraportal glucose and lipid infusions. If we did not observe fasting hyperglycemia, our innovative minipig model showed early signs of glucose intolerance and metabolic syndrome.

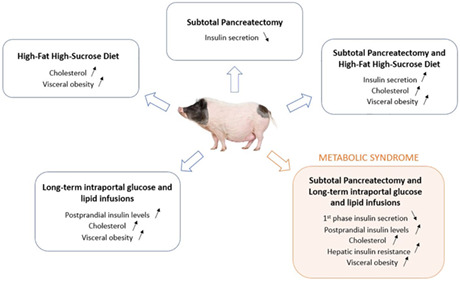

## 1 INTRODUCTION

Type 2 diabetes, one of the major diseases of the twenty‐first century, has an elevated prevalence (10.5% of the global population).^1^ The World Health Organization (WHO) defines this disease as reaching a glycemia over 126 mg/dL after 8 h of fasting, twice validated, or over 200 mg/dL following an oral glucose tolerance test.^2^ Currently, it is understood that a variety of pathophysiological processes plays a role in the onset of type 2 diabetes^1^, ^3^: decrease of insulin secretion, insulin resistance as a result of an imbalanced intake of carbohydrates and lipids and “thrifty genotype”.^4^ Additionally, type 2 diabetes is highly heterogenous and can be divided into five novel subtypes: severe autoimmune diabetes (related to type 1 diabetes), severe insulin‐deficient diabetes, severe insulin‐resistant diabetes, mild obesity related diabetes and mild‐age related diabetes.^5^, ^6^ Due to the disease's polymorphism, type 2 diabetes research needs the appropriate preclinical models in order to better understand pathophysiological pathways and create innovative, effective therapeutic approaches.

The most effective interventional treatment for type 2 diabetes in obese patients nowadays is metabolic surgery,^7^, ^8^, ^9^, ^10^ which enables an early diabetes remission independent of weight loss.^11^, ^12^, ^13^, ^14^ Current research on metabolic surgery is concentrated on understanding the link between the physiological changes that occur after intervention and the clinical benefit as well as on the development of new methods intended to improve the metabolic phenotype by reducing the associated complications.^15^ However, using preclinical models in which the surgical procedure may be easily applied to humans is one of the issues with metabolic surgery research. Porcine models have more translational value than rat models, even if rats are more often employed as preclinical models of metabolic surgery.^16^, ^17^, ^18^, ^19^ Pigs are in fact omnivorous and are similar to humans concerning the morphology and the physiology of their gastrointestinal tract, pancreas, propension to obesity and sedentary, metabolic biomarker levels and drug pharmacokinetics,^20^, ^21^ making it a particularly suitable preclinical model for these kinds of studies.

However, the creation of type 2 diabetes itself is the main problem with the preclinical pig model.

Current commercial pigs for meat production are the results of generations of selective breeding that targeted a phenotype able to store energy for later consumption by humans, likely making them protected against the deleterious effects of a “diabetogenic” environment.^22^ But some minipig strains, such as the Göttingen one, on which the physiology of insulin secretion is similar to humans,^23^, ^24^ or the Ossabaw, recognized to be a natural model of metabolic syndrome,^25^ were found to be more susceptible to metabolic issues. Because of this, various studies have attempted in the past to develop preclinical diabetic pig models from these strains. A number of strategies have been tried, including: a surgical strategy involving total or subtotal pancreatectomy, which results in a strong reduction in insulin secretion but has no effect on insulin sensitivity^26^, ^27^, ^28^; a chemical strategy involving the use of beta‐cell toxins, such as streptozotocin or alloxan, with variable results and significant hepato‐ and nephrotoxicity^29^, ^30^, ^31^, ^32^, ^33^; dietary interventions with a High‐Fat, High‐Sucrose diet, resulting in an insulin‐resistant and obesity‐related phenotype but not type 2 diabetes^34^, ^35^, ^36^, ^37^, ^38^; and genetic engineering, which can produce customized pig models^39^, ^40^, ^41^ but is logistically challenging and may have unintended consequences.^42^ In conclusion, no true type 2 diabetic pig with fasting hyperglycemia and insulin resistance conforming to type 2 diabetes definition has been created so far.

In the current study, we considered additional approaches involving the combination of existing methods: an oral energetic overload (a High‐Fat, High‐Sucrose diet) and a subtotal pancreatectomy were separately performed and then also combined, intended to outperform the pancreas's regulatory capabilities. A parenteral approach using long‐term intraportal glucose and lipid infusions, combined or not with a prior subtotal pancreatectomy, was also attempted. If the parenteral nutrition has already been set up to induce metabolic disorders in a piglet model,^43^, ^44^ it was, to our best knowledge, never tested in the adult pig as an approach to induce type 2 diabetes.

## 2 MATERIALS AND METHODS

### 2.1 Ethics statement

The local French Committee of Animal Research and Ethics (CEEA‐75, n°#18,915), in accordance with European law (2010/63/EU directive), accepted the protocol by approving it in accordance with the widely accepted ARRIVE guidelines. All the procedures were carried out in the agreed‐upon (n°D59‐35010) Département Hospitalo‐Universitaire de Recherche et d'Enseignement (Dhure) in the Faculty of Medicine in Lille, France.

### 2.2 Animals and housing

The study included a total of 21 healthy 1‐year‐old minipigs: 4 Ossabaw minipigs (DTU, Lyngby, Denmark) and 17 Göttingen‐like (Pannier, Wylder, France), weighing respectively 48.2 ± 1.9 kg and 31.7 ± 11.0 kg. Our local minipig strain, called Göttingen‐like, was created more than 30 years ago as a consequence of an initial crossing with the Göttingen strain. To limit the metabolic differences related to the female hormonal cycle, only males were included. At the start of the protocol, animals were either surgically castrated or delivered castrated in the animal facility. All animals were housed and enriched in individual boxes in conventional conditions. Water was provided ad libitum and 400 g of standard food (Swine Engrais‐F S25/T, Uneal Cooperative) was given twice a day. The composition of the standard diet was detailed in Table 1. The light/dark cycle was 12 h of light and 12 h of darkness with a temperature between 19 and 24°C. Pigs benefited from a 15‐day acclimatization period.

**TABLE 1:** Composition (in %) of the standard diet and the High‐Fat High‐Sucrose (HFHS) diet given to the minipigs.

Composition	Standard diet	HFHS diet
Wheat	10.00	6.25
Barley	34.00	12.00
Wheat bran	25.00	11.14
Soybean cake	6.00	12.00
Sunflower cake	10.00	8.00
Soybean hulls	12.00	8.86
Cornstarch		6.50
Saccharose		20.00
Calcium carbonate	1.30	1.30
Sodium phosphate	0.60	0.60
Sodium chloride	0.60	0.60
Vitamins and minerals	0.50	0.75
Lard		12.00
**Energetic density**	12.5 kJ/g	20.9 kJ/g

### 2.3 Study design

**FIGURE 1:**
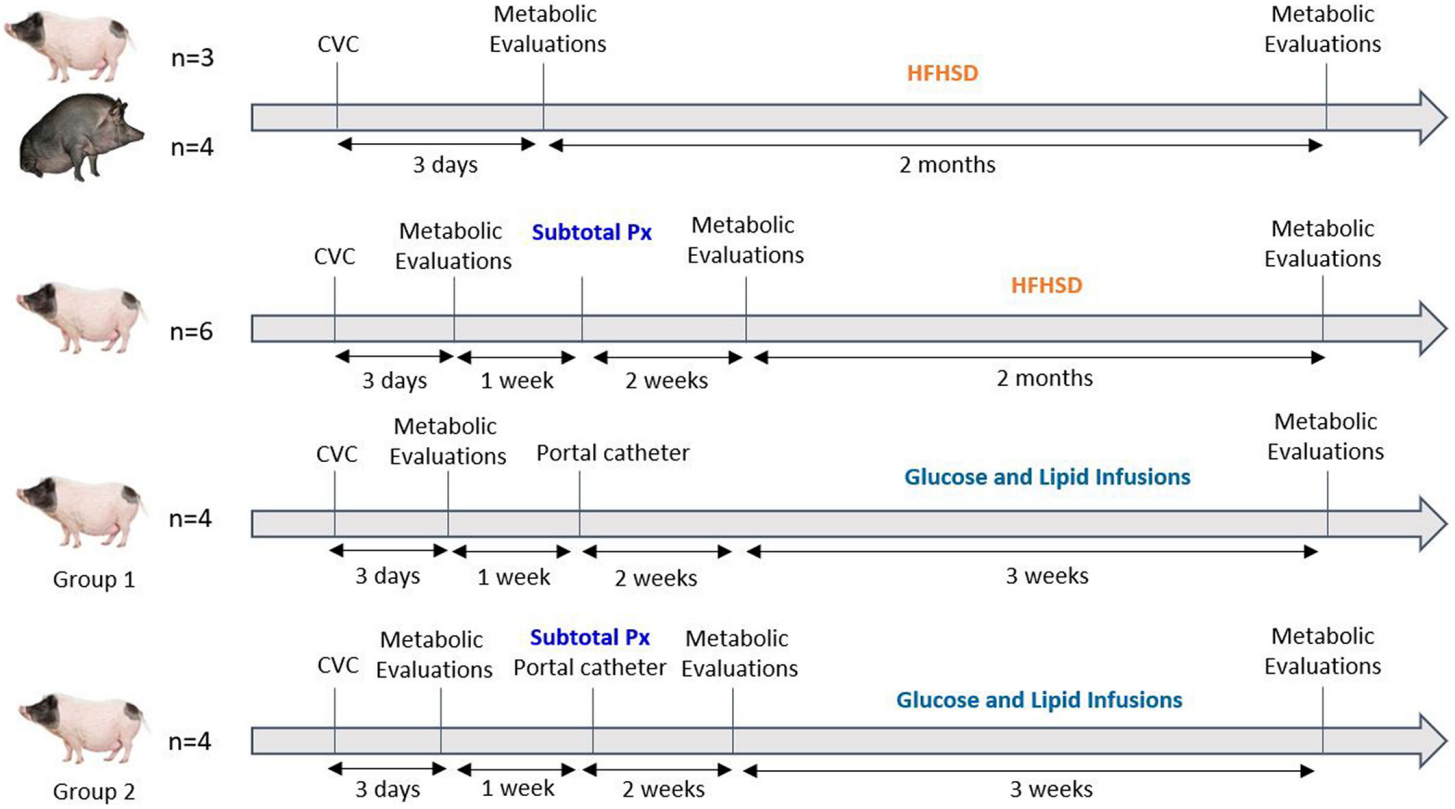
Overall study design for each group of animals. CVC, Central Venous Catheter; HFHSD, High‐Fat High‐Sucrose diet; Px, Pancreatectomy. The fully black minipig represents the Ossabaw strain, while the pink and black ones indicate the Göttingen‐like strain. A Mixed Meal Test (MMT) and an Intravenous Glucose Tolerance Test constitute metabolic assessments (IVGTT). Metabolic tests were performed on all minipigs (*n* = 10) with subtotal pancreatectomy 2 weeks after the intervention.

In this study, we wanted to create a preclinical pig model of type 2 diabetes that corresponds to the WHO definition.^2^ This led to the combination of a partial pancreatectomy, a surgical method of insulin deprivation, with methods of energetic overload, via oral or intraportal administration. In order to determine which strain of minipigs was most suited for our strategy, we first assessed how both the Göttingen‐like strain (*n* = 3) and the Ossabaw one (*n* = 4) responded to a 2‐month High‐Fat, High‐Sucrose diet (HFHSD). We decided thereafter to discard the Ossabaw strain because Göttingen‐like showed a phenotype closer to our expectations. Following this choice, we combined a subtotal pancreatectomy with 2 months of HFHSD in Göttingen‐like minipigs (*n* = 6). Finally, we explored a different strategy by infusing intraportal glucose and lipids for 3 weeks as a parenteral energetic overload in two groups of Göttingen‐like minipigs: in Group 1 (*n* = 4), no subtotal pancreatectomy was initially performed; in Group 2 (*n* = 4), a subtotal pancreatectomy was performed prior to the energetic overload. The impact of the subtotal pancreatectomy on glucose metabolism in the Göttingen‐like strain was simultaneously examined (*n* = 10), including the animals subjected to the HFHSD following the pancreatectomy (*n* = 6) and those of Group 2 (*n* = 4). Figure 1 displays the general design of this research.

### 2.4 Surgical procedures

#### 2.4.1 Anaesthesia and analgesia

Following an overnight fast, all surgical procedures were performed under general anaesthesia. Premedication included intramuscular injections of xylazine (3 mg/kg; Sedaxylan®; Dechra Pharmaceutical PLC, France) and ketamine (5 mg/kg; Ketamine 1000®; Virbac, France), followed by isoflurane after endotracheal intubation (0.5 to 2%; IsoFlo; Zoetis, France). During the laparotomy procedures, animals were ventilated with assistance at 20 mpm or left with spontaneous ventilation. To ensure analgesia, an intramuscular injection of buprenorphine (15 μg/kg, Bupaq®, Virbac, France) for the insertion of a central venous catheter or a single transdermal application of fentanyl (1.3 mg/kg, Recuvyra®, Lilly‐Elanco, France) for laparotomy procedures were used.^45^

#### 2.4.2 Implantation of a central venous catheter (CVC)

The external jugular vein was exposed in the neck region after skin and muscle incision. After venotomy, the catheter (Hickman® 9.6F Single‐Lumen CV Catheter, Bard Access System, USA) was inserted and linked to the vein with two ligatures (Vicryl® Bobine 2/0, Ethicon, France). It was tunnelized via the subcutaneous tissue from the incision zone to the dorsal area of the neck. Muscular and cutaneous layers were then closed by simple overlock (respectively Polysorb® 2/0, Medtronic, France and Mersilene® 1, Ethicon, France). This catheter remained throughout the duration of the procedure and was kept operational by administering 5 mL of physiological serum that had been heparinized (1 mL heparin at 5000 IU/mL for 250 mL NaCl 0.9%) after each usage or every 2 days if it was not.

#### 2.4.3 Subtotal pancreatectomy

By reclining the stomach cranially and the intestinal system caudally, the pancreas was made accessible. From the tail to the head, the dissection was carried out (splenic lobe). In the retro‐portal region, the connecting lobe was similarly dissected and largely removed. Before section and extraction, ligatures between the splenic and the duodenal lobes were performed, and the connecting lobe was also tightened. As previously stated,^46^ the subtotal pancreatectomy involved removing 75% of the total organ weight.

#### 2.4.4 Portal catheter implantation

The spleen was removed from the abdominal cavity after median laparotomy of the supra ombilical region, and the splenic vein was dissected on 2 cm. On the left flank, behind the final rib, the catheter (Hickman® 9.6F Single‐Lumen CV Catheter, Bard Access System, USA) was tunnelized across the abdominal wall. The catheter was placed following the splenic vein venotomy, advanced through the spleno‐mesaraic confluence into the portal vein, and secured to the splenic vein with two ligatures. The layers of the peritoneum, muscles and skin were closed by a simple overlock.

#### 2.4.5 Surgical castration

Medially between the scrotum and the penile region, cutaneous and subcutaneous tissues were incised after testicles were compressed cranially. Additionally, the tunica vaginalis was cut open to reveal the testis. The cauda epididymis ligament was cut after extraction. Two ligatures were used to ligate the spermatic lead, and it was then sectioned. Simple overlock was used to seal the tunica vaginalis and scrotum.

### 2.5 Energetic overload

#### 2.5.1 High‐Fat High‐Sucrose diet (HFHSD)

Animals were fed with a HFHSD for 2 months. The Institut National de Recherche pour l'Agriculture, l'Alimentation et l'Environnement (Inrae, France) determined the food's composition. Seven hundred and fifty grams of HFHSD were administered twice a day and contained 61.7% carbohydrates, 23.2% fats, and 15.1% proteins. The composition of the HFHS diet was detailed in Table 1.

#### 2.5.2 Intraportal glucose and lipid infusions

Over the period of 3 weeks, the intraportal catheter was used to administer lipid and glucose infusions twice daily for 2 h. A 2‐h gap between the bi‐daily infusions was observed. Fifty percent glucose (G50®, B. Braun, France) and lipid solution (Intralipid20®, Fresenius Kabi, France) was administered using infusion pumps (SK 600II®, Mano Medical, France) at respective flow rates of 125 mL/h and 63 mL/h. These flow rates were selected in order to maintain each infusion's glycemia above 500 mg/dL. Each infusion was preceded by a 500 mg/kg bolus of 50% glucose solution to raise blood glucose levels to more than 500 mg/dL within 1 min. All the infusions were performed in an awake animal.

### 2.6 Metabolic tests

#### 2.6.1 Mixed meal tests (MMT)

After a 12‐h fast, a 20‐g solid energy bar (Ovomaltine®, Nestlé, France) and 200 mL of liquid (Fortimel Energy®, Nutricia, France) were mixed and given vigil via a nasogastric tube of 16 Fr that had previously been implanted under general anaesthesia during the CVC implantation procedure for the first MMT or the day before for the other MMT. The meal had a 990‐kJ energy density and contained 49 g of total carbohydrates, 13 g of fats, and 15 g of proteins. On EDTA and heparinised tubes, blood samples were obtained before the MMT was administered (*t* = 0 min) and at various time intervals afterwards (*t* = 15, *t* = 30, *t* = 60, *t* = 90, *t* = 120, and *t* = 180 min).

#### 2.6.2 Intravenous glucose tolerance test (IVGTT)

Following an overnight fast, a 50‐% glucose solution (G50®, B. Braun, France) was intravenously administered into the CVC at a dose of 500 mg/kg. On EDTA tubes, blood samples were taken in the awake animal before (*t* = 0 min) and following the administration of glucose at *t* = 1, *t* = 3, *t* = 5, *t* = 10, *t* = 15, and *t* = 30 min.

Plasma was collected from each tube, centrifuged at 4000 rpm for 10 min at 4°C, and then stored at −80°C until analyses.

### 2.7 Biological analyses

The amperometric glucose oxidase method was used to measure the level of glucose in blood (glucometer Accu‐Chek Performa®, Roche, France, or Nova Biomedical StatStrip Xpress®, DSI, USA). A DXI Access Immunoassay System (Beckman Coulter) with an assay range between 0.3 and 300 μIU/mL was used to measure the plasma insulin concentrations, as previously mentioned.^47^ Plasma lipid profile (total cholesterol, LDL, HDL and triglycerides) was assessed using an Abbott Architect C4000® clinical chemistry analyser.

### 2.8 Calculations and statistical analyses

For data analysis, GraphPad Prism v8® software was employed. For curves, the results were expressed as mean ± SEM, and for histograms, as mean ± SD. Depending on the situation, paired or unpaired Student's *t*‐tests were used to analyse the variables. A Two‐Way ANOVA and Sidak post‐hoc tests were used to compare blood glucose and plasma insulin levels during the MMT and IVGTT between the different strains of minipigs or between baseline and after diabetogenic interventions. For each comparison of blood glucose or insulin evolution during metabolic test, the effect of time of the metabolic test (called “time”) and strain (Göttingen‐like or Ossabaw, called “strain”) or diabetogenic intervention (“HFHSD”, “pancreatectomy”, or “infusions”) was systematically assessed. The presence of interaction between “time” and “strain” or “time” and “intervention” was also evaluated. The calculation of Insulinogenic Index was performed to evaluate the postprandial early insulin secretion as described^48^: [Plasma Insulin (*t* = 30)–Plasma Insulin (*t* = 0)]/[Blood Glucose (*t* = 30)–Blood Glucose (*t* = 0)], with plasma insulin in μIU/mL and blood glucose in mg/dL. The hepatic insulin resistance was evaluated thanks to the Hepatic Insulin Resistance Index (HIRI) calculation as previously described,^49^ by multiplication of the first 30‐min area under the curve between glucose and insulin concentration during MMT. The Acute Insulin Response (AIR), which describes the initial phase of insulin production following intravenous glucose stimulation, was computed by subtracting fasting insulin levels from the mean evaluation of plasma insulin levels at 1, 3 and 5 min.^50^, ^51^

## 3 RESULTS

### 3.1 Choice of the Göttingen‐like minipig strain after comparison with Ossabaw

**FIGURE 2:**
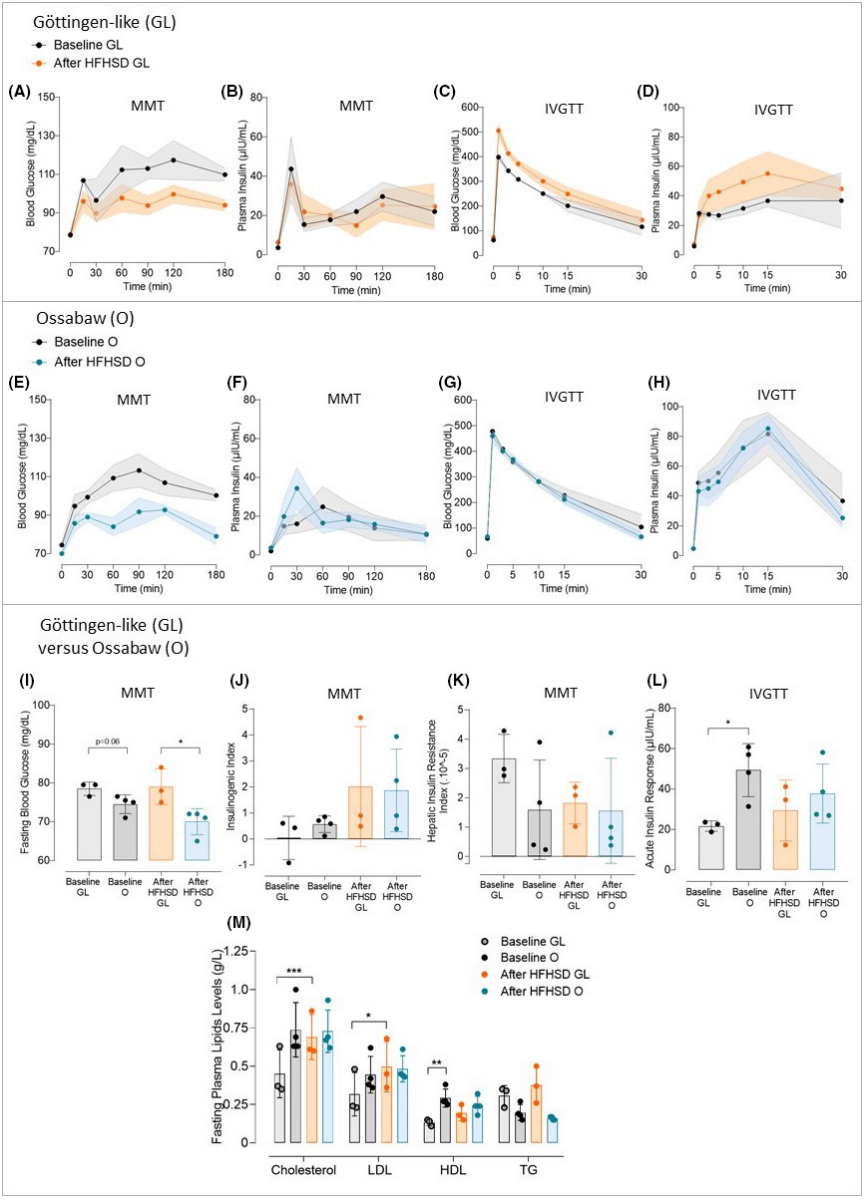
Comparison of the Ossabaw and Göttingen‐like minipig strains' metabolic responses to a 2‐month High‐Fat, High‐Sucrose diet. (A–D) Mean curves (Mean ± SEM; *n* = 3) of Blood Glucose (A) and Plasma Insulin (B) during Mixed Meal Test (MMT) and Blood Glucose (C) and Plasma Insulin (D) during Intravenous Glucose Tolerance Test (IVGTT) in the Göttingen‐like (GL) strain, with the baseline characteristics represented in black and after 2‐month High‐Fat High‐Sucrose diet (HFHSD) in orange. (E–H) Mean curves (Mean ± SEM; *n* = 4) of Blood Glucose (E) and Plasma Insulin (F) during MMT and Blood Glucose (G) and Plasma Insulin (H) during IVGTT in the Ossabaw (O) strain, with the baseline characteristics represented in black and after HFHSD in blue. (I) Mean Fasting Blood Glucose (Mean ± SD) measured during MMT before (in black) and after (in colour) HFHSD in the Göttingen‐like (GL, in orange; *n* = 3) and in the Ossabaw (O, blue; *n* = 4) strains. (J, K) Mean Insulinogenic Index (J) and Hepatic Insulin Resistance Index (K) (Mean ± SD) calculated during MMT before (in black) and after (in colour) HFHSD in the Göttingen‐like (GL, in orange; *n* = 3) and in the Ossabaw (O, blue; *n* = 4) strains. (L) Mean Acute Insulin Response (Mean ± SD) calculated during IVGTT before (in black) and after (in colour) HFHSD in the Göttingen‐like (GL, in orange; *n* = 3) and in the Ossabaw (O, blue; *n* = 4) strains. (M) Fasting plasma lipid profile (Mean ± SD) assessed before (in black) and after (in colour) HFHSD in the Göttingen‐like (GL, in orange; *n* = 3) and in the Ossabaw (O, blue; *n* = 4) strains. HDL, high‐density lipoprotein; LDL, low‐density lipoprotein; TG, triglycerides; Total Chol, total cholesterol. Two‐Way ANOVA test for repeated measures and Sidak post‐hoc test; Paired or unpaired *t*‐test; *p < .05, **p < .01, ***p < .001.

The glucose metabolism of Göttingen‐like (GL, *n* = 3) and Ossabaw (O, *n* = 4) minipigs was compared before and after a 2‐month High‐Fat High‐Sucrose diet (HFHSD) in order to determine which strain was best suited for our procedure (Figure 2). Following the regimen, both strains notably gained weight (55.1 ± 4.3 after vs. 43.4 ± 6.4 kg before for GL, *p* < .05 and 62.8 ± 4.8 after vs. 48.2 ± 1.9 kg before for O, *p* < .01), corresponding to a weight gain of 26.9% for GL and 30.3% for O. Postprandial blood glucose concentrations (Figure 2A) were generally lower but not significantly following HFHSD, while insulin concentrations (Figure 2B) did not differ in Göttingen‐like minipigs. The intravenous glucose tolerance test (IVGTT) results for glycemia (Figure 2C) and insulin (Figure 2D) did not change between the two steps. After HFHSD, Ossabaw minipigs showed a trend of lower postprandial blood glucose levels (Figure 2E) accompanied by a trend of higher insulin peak secretion (Figure 2F). However, during the IVGTT, there were no discernible changes between the glucose decline (Figure 2G) and corresponding insulin concentrations (Figure 2H). Ossabaw minipigs' fasting blood glucose appeared lower than Göttingen‐like ones at baseline, and it was significantly lower after HFHSD than those of Göttingen‐like (70.0 ± 3.4 after vs. 79.0 ± 4.6 mg/dL before; *p* < .05) (Figure 2I). Figure 2J shows a trend of increasing Insulinogenic Index for both strains after HFHSD, although there was no change in Hepatic Insulin Resistance Index (Figure 2K). Compared to Göttingen‐like minipigs, Ossabaw minipigs had significantly higher baseline Acute Insulin Response (49.3 ± 13.1  μIU/mL for O vs. 21.7 ± 2.5 μIU/mL for GL, *p* < .05) although no discernible alterations were found for any strain following HFHSD (Figure 2L). Ossabaw minipigs presented a higher level in fasting cholesterol at the baseline than Göttingen‐like minipigs (Figure 2M) (total cholesterol: 0.74 ± 0.18 g/L for O vs. 0.45 ± 0.16 g/L for GL, not significant; LDL: 0.45 ± 0.12 g/L for O vs. 0.32 ± 0.14 g/L for GL, not significant; HDL: 0.29 ± 0.06 g/L for O vs. 0.13 ± 0.02 g/L for GL, *p* < .01). However, there was no change in the lipid profile after HFHSD in Ossabaw minipigs while total cholesterol and LDL levels were significantly increased in Göttingen‐like minipigs (total cholesterol: 0.69 ± 0.15 g/L after vs. 0.45 ± 0.16 g/L before, *p* < .001; LDL: 0.50 ± 0.17 g/L after vs. 0.32 ± 0.14 g/L before, *p* < .05). These findings indicated that Ossabaw minipigs had a better early insulin response than Göttingen‐like minipigs. We thus decided to proceed with our strategy using the Göttingen‐like strain.

### 3.2 Reduction of acute insulin response after subtotal pancreatectomy in Göttingen‐like minipigs

**FIGURE 3:**
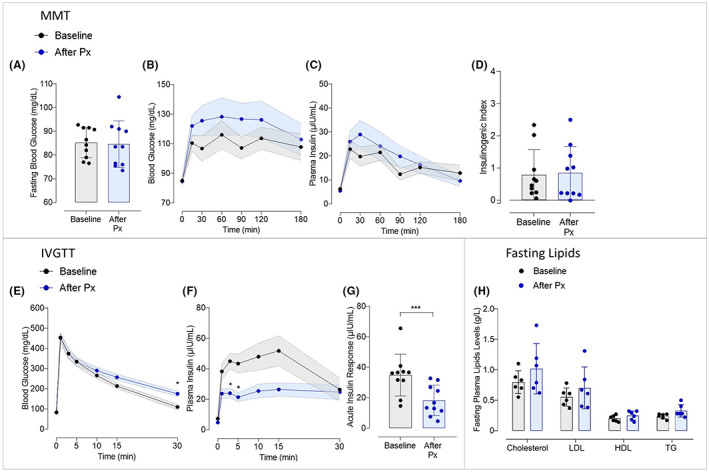
Evaluation of the effect of a subtotal pancreatectomy on glucose metabolism in Göttingen‐like minipigs. (A) Mean Fasting Blood Glucose (Mean ± SD; *n* = 10) measured during MMT before (in black) and after (in blue) subtotal pancreatectomy. (B, C) Mean curves (Mean ± SEM; *n* = 10) of Blood Glucose (B) and Plasma Insulin (C) during MMT before (in black) and after (in blue) subtotal pancreatectomy. (D) Mean Insulinogenic Index (Mean ± SD; *n* = 10) calculated during MMT before (in black) and after (in blue) subtotal pancreatectomy. (E, F) Mean curves (Mean ± SEM; *n* = 10) of Blood Glucose (E) and Plasma Insulin (F) during IVGTT before (in black) and after (in blue) subtotal pancreatectomy. (G) Mean Acute Insulin Response (Mean ± SD; *n* = 10) calculated during IVGTT before (in black) and after (in blue) subtotal pancreatectomy. (H) Fasting plasma lipid profile (Mean ± SD; *n* = 10) assessed before (in black) and after (in blue) subtotal pancreatectomy. HDL, high‐density lipoprotein; LDL, low‐density lipoprotein; TG, triglycerides; Total Chol, total cholesterol. Two‐Way ANOVA test for repeated measures and Sidak post‐hoc test; Paired *t*‐test; *p < .05, ***p < .0005 between baseline and after pancreatectomy.

We assessed how a subtotal pancreatectomy affected glucose metabolism (Figure 3). After the surgical procedure, there was no rise in fasting blood glucose (Figure 3A). Mixed Meal Tests did not reveal any appreciable changes in blood glucose (Figure 3B) or insulin levels (Figure 3C). As a result, there was no change in the Insulinogenic Index (Figure 3D). When compared to before the intervention, the speed at which the glucose levels declined during the IVGTT following pancreatectomy was slower (blood glucose levels of respectively 175.1 ± 12.4 mg/dL after vs. 109.4 ± 13.1 mg/dL before at 30 min, *p* < .05) (Figure 3E). A significant interaction between “time” and “pancreatectomy” was thus reported (*p* < .05). Plasma insulin levels during IVGTT were significantly lower after pancreatectomy than at the baseline and especially at 3 and 5 min (respectively 24 ± 3.2 μIU/mL after vs. 45 ± 5.1 μIU/mL before and 21 ± 2.8 μIU/mL after vs. 43 ± 6.1 μIU/mL, p < .05) (Figure 3F). A significant interaction between “time” and “pancreatectomy” was thus noticed (*p* < .0001). As a result, following pancreatectomy, the Acute Insulin Response was significantly decreased (18.3 ± 10.0 μIU/mL after vs. 34.9 ± 13.7 μIU/mL before, *p* < .0005) (Figure 3G). Finally, there was no significant change reported in fasting plasma lipid profile after subtotal pancreatectomy (Figure 3H).

### 3.3 No significant change in glucose metabolism following the combination of a subtotal pancreatectomy with a 2‐month HFHSD in Göttingen‐like minipigs

**FIGURE 4:**
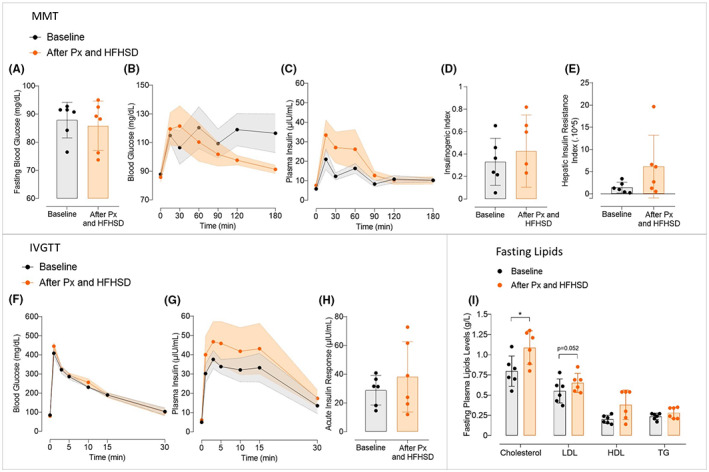
Effect of the combination of a subtotal pancreatectomy followed by a 2‐month High‐Fat High‐Sucrose diet on glucose metabolism in Göttingen‐like minipigs. (A) Mean Fasting Blood Glucose (Mean ± SD; *n* = 6) measured during MMT before (in black) and after (in orange) subtotal pancreatectomy followed by a 2‐month High‐Fat High‐Sucrose diet (HFHSD). (B, C) Mean curves (Mean ± SEM; *n* = 6) of Blood Glucose (A) and Plasma Insulin (B) during MMT before (in black) and after (in orange) subtotal pancreatectomy followed by a 2‐month HFHSD. (D, E) Mean Insulinogenic Index (D) and Hepatic Insulin Resistance Index (E) (Mean ± SD; n = 6) calculated during MMT before (in black) and after (in orange) subtotal pancreatectomy followed by a 2‐month HFHSD. (F, G) Mean curves (Mean ± SEM; *n* = 6) of Blood Glucose (F) and Plasma Insulin (G) during IVGTT before (in black) and after (in orange) subtotal pancreatectomy followed by a 2‐month HFHSD. (H) Mean Acute Insulin Response (Mean ± SD; *n* = 6) calculated during IVGTT before (in black) and after (in orange) subtotal pancreatectomy followed by a 2‐month HFHSD. (I) Fasting plasma lipid profile (Mean ± SD; *n* = 6) assessed before (in black) and after (in orange) subtotal pancreatectomy followed by a 2‐month HFHSD. HDL, high‐density lipoprotein; LDL, low‐density lipoprotein; TG, triglycerides; Total Chol, total cholesterol. Two‐Way ANOVA test for repeated measures and Sidak post‐hoc test; Paired *t*‐test; *p < .05.

The metabolic phenotypic changes following a subtotal pancreatectomy and 2 months of HFHSD as an oral energy overload were then examined (Figure 4). Following the protocol, animals gained weight (26.3 ± 5.9 kg after, compared to 21.3 ± 3.6 kg before, *p* < .05). Following this approach, no rise in fasting blood glucose (Figure 4A) was observed. With a more pronounced peak at 30 min and a faster return to baseline following the procedure, postprandial blood glucose dynamics were different from before, even if not significantly (Figure 4B). Although there was a trend to higher postprandial insulin levels (Figure 4C), the Insulinogenic Index did not significantly change (Figure 4D). Additionally, the Hepatic Insulin Resistance Index modestly but not significantly increased (Figure 4E). The IVGTT revealed no significant changes in glucose tolerance (Figure 4F), insulin levels (Figure 4G), or Acute Insulin Response (Figure 4H). Finally, the levels of fasting plasma lipids were globally increased after intervention (Figure 4I) (total cholesterol: 1.09 ± 0.20 g/L after vs. 0.80 ± 0.19 g/L before, *p* < .05; LDL: 0.65 ± 0.12 g/L after vs. 0.55 ± 0.15 g/L before, *p* = .052; HDL: 0.38 ± 0.18 g/L after vs. 0.20 ± 0.05 g/L before, not significant; triglycerides: 0.28 ± 0.07 g/L after vs. 0.24 ± 0.04 g/L before, not significant).

### 3.4 Alterations of insulin secretion pattern and insulin resistance after long‐term intraportal glucose and lipid infusions in Göttingen‐like minipigs

**FIGURE 5:**
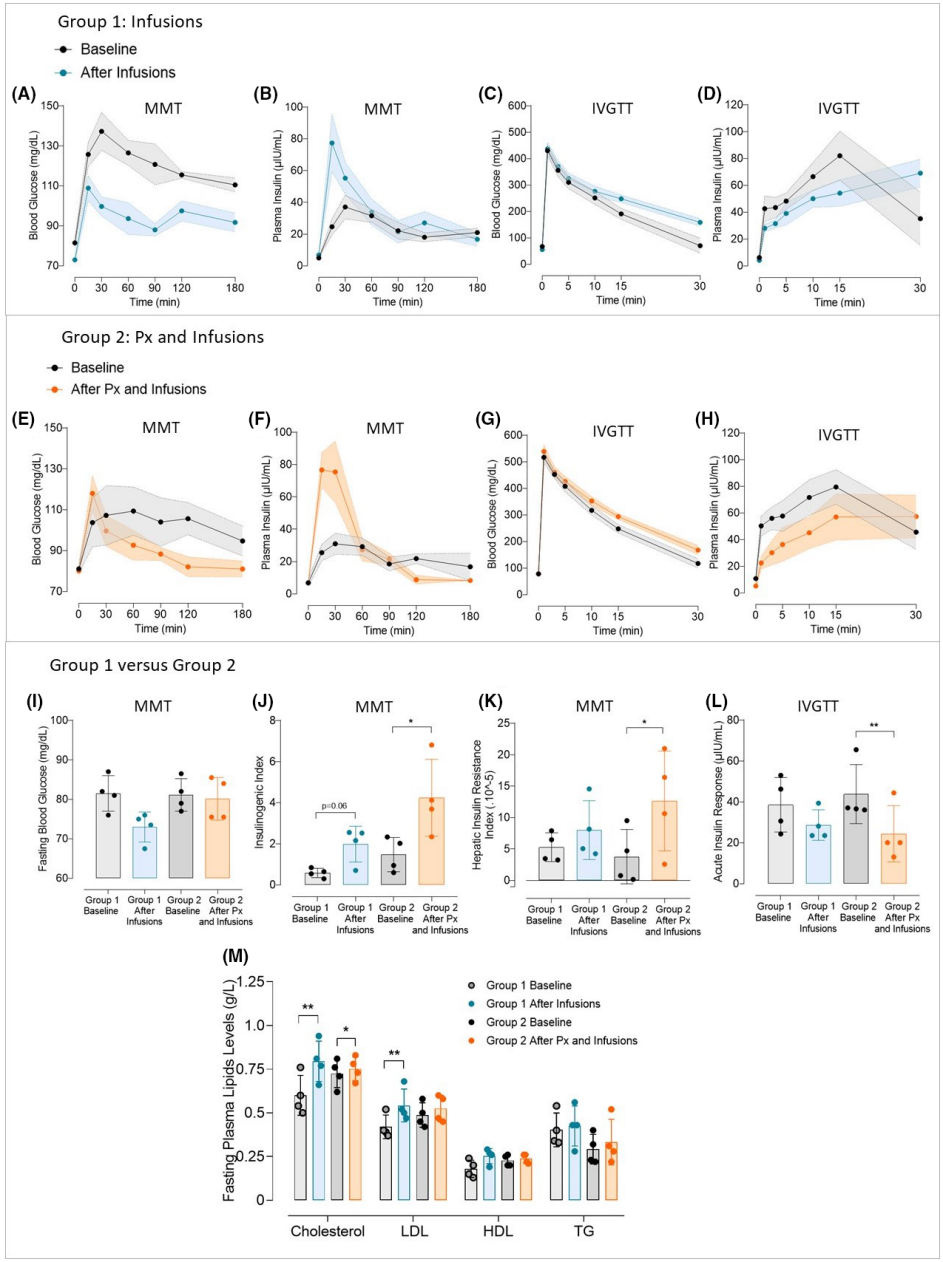
Effect of long‐term intraportal glucose and lipid infusions on glucose metabolism in Göttingen‐like minipigs, whether or not they are preceded by a subtotal pancreatectomy. (A–D) Mean curves (Mean ± SEM; *n* = 4) of Blood Glucose (A) and Plasma Insulin (B) during Mixed Meal Test (MMT) and Blood Glucose (C) and Plasma Insulin (D) during Intravenous Glucose Tolerance Test (IVGTT) before (in black) and after (in blue) 3 weeks of long‐term intraportal glucose and lipids infusions. (E–H) Mean curves (Mean ± SEM; *n* = 4) of Blood Glucose (E) and Plasma Insulin (F) during Mixed Meal Test (MMT) and Blood Glucose (G) and Plasma Insulin (H) during Intravenous Glucose Tolerance Test (IVGTT) before (in black) and after (in orange) the combination of a subtotal pancreatectomy followed by 3 weeks of long‐term intraportal glucose and lipid infusions. (I) Mean Fasting Blood Glucose (Mean ± SD; *n* = 4 per group) measured during MMT at the baseline for Group 1 (in light grey) and Group 2 (in dark grey) and after 3 weeks of long‐term intraportal glucose and lipid infusions (Group 1, in blue) and after subtotal pancreatectomy followed by 3 weeks of long‐term intraportal glucose and lipid infusions (Group 2, in orange). (J, K) Mean Insulinogenic Index (J) and Hepatic Insulin Resistance Index (K) (Mean ± SD; *n* = 4 per group) calculated during MMT at the baseline for Group 1 (in light grey) and Group 2 (in dark grey) and after 3 weeks of long‐term intraportal glucose and lipid infusions (Group 1, in blue) and after subtotal pancreatectomy followed by 3 weeks of long‐term intraportal glucose and lipid infusions (Group 2, in orange). (L) Mean Acute Insulin Response (Mean ± SD; *n* = 4 per group) calculated during IVGTT at the baseline for Group 1 (in light grey) and Group 2 (in dark grey) and after 3 weeks of long‐term intraportal glucose and lipid infusions (Group 1, in blue) and after subtotal pancreatectomy followed by 3 weeks of long term intraportal glucose and lipid infusions (Group 2, in orange). (M) Fasting plasma lipid profile (Mean ± SD; *n* = 4 per group) assessed at the baseline for Group 1 (in light grey) and Group 2 (in dark grey) and after 3 weeks of long‐term intraportal glucose and lipid infusions (Group 1, in blue) and after subtotal pancreatectomy followed by 3 weeks of long‐term intraportal glucose and lipid infusions (Group 2, in orange). HDL, high‐density lipoprotein; LDL, low‐density lipoprotein; TG, triglycerides; Total Chol, total cholesterol. Two‐Way ANOVA test for repeated measures and Sidak post‐hoc test; Paired *t*‐test; *p < .05, **p < .01.

In two groups of minipigs, one without prior pancreatectomy and the other following subtotal pancreatectomy, we infused long‐term intraportal glucose and lipid (Figure 5). Animals of each group gained a little weight following infusions (35.2 ± 11.4 after vs. 28.3 ± 7.4 kg before for Group 1, *p* < .05; and 47.4 ± 6.5 kg after vs. 41.8 ± 6.7 kg before for Group 2, *p* < .005). Postprandial blood glucose levels of Group 1 were significantly lower following infusions compared to the baseline state (*p* < .005) (Figure 5A). A significant interaction between “time” and “infusions" was thus noticed (*p* < .05). The first 30‐min showed a rise in plasma insulin levels (77.4 ± 18.0 μIU/mL after infusions at 15 min vs. 24.7 ± 5.1 μIU/mL before, and 55.3 ± 10.0 μIU/mL after infusions at 30 min vs. 37.1 ± 7.6 μIU/mL before; not significant) and a significant interaction between “time” and “infusions” was discovered (*p* < .005) (Figure 5B). Blood glucose levels decreased during IVGTT more slowly than they did before protocol (159.0 ± 14.2 mg/dL after infusions vs. 70.4 ± 28.6 mg/dL before at 30 min; not significant) (Figure 5C) and insulin levels globally decreased, with an exception at 30 min (Figure 5D). Following procedure, postprandial blood glucose levels in Group 2 fell globally (Figure 5E), similar to Group 1 and a significant interaction between “time” and “intervention” was observed (*p* < .05). Plasma insulin levels rose for the first 30 min (76.7 ± 10.6 μIU/mL after protocol at 15 min vs. 25.5 ± 5.1 μIU/mL before, and 75.4 ± 19.0 μIU/mL after protocol at 30 min vs. 31.0 ± 6.5 μIU/mL before; not significant) and a significant interaction between “time” and “intervention” was reported (*p* < .0001) (Figure 5F). IVGTT findings after protocol revealed a slower lowering of blood glucose (Figure 5G) and especially lower insulin levels with a significant intervention observed between “time” and “intervention” (*p* < .05) (Figure 5H). Finally, whether or not a subtotal pancreatectomy had been performed prior to the intraportal glucose and lipid infusion, no increase in fasting blood glucose was observed (Figure 5I). However, both groups showed an increase in the Insulinogenic Index (2.0 ± 0.8 vs. 0.59 ± 0.2; *p* = .06 and 4.2 ± 1.9 vs. 1.5 ± 0.8; *p* < .05, respectively for Groups 1 and 2) (Figure 5J). An increase in Hepatic Insulin Resistance Index (×10^−5^) was also obtained (8.0 ± 4.7 after vs. 5.3 ± 2.3 before; not significant, and 12.6 ± 7.9 after vs. 3.8 ± 4.3 before; *p* < .05, respectively for Groups 1 and 2) (Figure 5K). Additionally, both groups' Acute Insulin Responses reduced (28.7 ± 7.5 µIU/mL after vs. 38.6 ± 13.3 µIU/mL before; not significant, and 24.4 ± 13.7 µIU/mL after vs. 43.9 ± 14.5 µIU/mL before; *p* < .005 before, respectively for Groups 1 and 2) (Figure 5L). Finally, fasting plasma levels of total cholesterol and LDL were increased after intervention for both groups (Figure 5M) (total cholesterol: 0.80 ± 0.12 g/L after vs. 0.60 ± 0.11 g/L before, *p* < .01, for Group 1 and 0.75 ± 0.07 g/L after vs. 0.73 ± 0.08 g/L before, *p* < .05, for Group 2; LDL: 0.54 ± 0.09 g/L after vs. 0.42 ± 0.07 g/L before, *p* < .01, for Group 1 and 0.53 ± 0.08 g/L after vs. 0.49 ± 0.07 g/L before, not significant, for Group 2). HDL and triglycerides levels were not significantly altered after intervention, no matter the group. No significant difference was observed in glucose homeostasis and lipid profile after intervention between Group 1 and Group 2.

## 4 DISCUSSION

We attempted to develop a preclinical type 2 diabetic pig model in this study in accordance with the World Health Organization definition (glycemia over 126 mg/dL after 8 h of fasting, verified twice, or over 200 mg/dL following an oral glucose tolerance test).^2^ In order to determine which strain of pigs was the most suited, we first subjected two distinct strains to a High‐Fat High‐Sucrose diet (HFHSD) for 2 months. This enabled us to evaluate each strain's metabolic adaptation to the HFHSD. Both strains responded equally, with Ossabaw having a little better insulin response than Göttingen‐like, which justified pursuing the study with Göttingen‐like minipigs. During metabolic evaluations, the metabolic response in both strains showed a tendency to an increase in insulin levels, necessitating the introduction of an intervention aimed at reducing the capacity of pancreas adaptability. This is why we decided to combine an energy overload with an insulin restriction technique, such as a subtotal pancreatectomy. In the beginning, the effects of a single subtotal pancreatectomy were investigated. In our investigation, subtotal pancreatectomy reduced early insulin secretion while leaving postprandial glycemic response and fasting glycemia unaffected. The subtotal pancreatectomy was performed on a set of pigs combined with 2 months of oral energy overload administered via a HFHSD: there was no change in the metabolism of glucose. Thus, another strategy was considered in moving towards a parenteral energetic overload using chronic intraportal glucose and lipid infusions, whether or not they were associated with a preceding subtotal pancreatectomy. Results showed similar patterns with or without a pancreatectomy, with lower postprandial glycemia values associated with a higher 30‐min insulin peak. A rise in hepatic insulin resistance was also observed, particularly in the group that was subjected to the subtotal pancreatectomy prior to infusions, in addition to this postprandial hyperinsulinism. Finally, during intravenous glucose tolerance testing, a decrease in the first phase of insulin secretion was observed for both groups, with the pancreatectomy group showing significant differences. We were unable to produce a type 2 diabetic minipig model because none of the study groups achieved a fasting hyperglycemia.

The results in the Ossabaw strain were unexpected. The HFHSD induced a response that was highly comparable to that of the Göttingen‐like strain, with an early insulin secretion that appeared to be even more effective than the Göttingen one in the baseline state. However, Ossabaw minipigs have a reputation for being the strain that is most susceptible to metabolic syndrome.^25^, ^52^ In fact, they developed, in the “Ossabaw Georgia island” where they come from, a “thrifty genotype” that enabled them to easily store energy from low‐nutritive substrates because of the severe selection pressure imposed by the dry climate of the Ossabaw island. Thus, it is claimed that Ossabaw minipigs serve as a natural model for reproducing the symptoms of type 2 diabetes and the metabolic syndrome, similar to those populations that are predisposed to these diseases naturally.^3^ However, given that no fasting hyperglycemia could be generated only after a diet in previous research,^53^, ^54^, ^55^ it would appear that the expression of their metabolic syndrome would be more focused on lipidic dysregulations than disorders of glucose metabolism.^56^, ^57^ Fasting lipid levels of Ossabaw minipigs were much greater than those of the Göttingen‐like strain in our study, particularly in terms of cholesterol, which makes this strain well‐suited for the investigation of hypercholesterolemia illnesses^58^ but not for studies of diabetes. We continued the combination protocol, which included a subtotal pancreatectomy, followed by five more months of HFHSD, in two minipigs of this strain. These two minipigs showed no signs of metabolic change (data not shown), demonstrating that this strain is not susceptible to develop type 2 diabetes.

The decision to perform a pancreatectomy was made considering the highly variable and toxic effects of streptozotocin^59^ and alloxan.^60^ Additionally, a surgical pancreatic mass excision is easier to control than one caused by toxic chemicals,^42^, ^46^ which is why this way of generating an insulin deficiency was chosen. The partial pancreatectomy's subsequent impact on glucose metabolism was unexpectedly modest, with the only discernible change being a reduction in the acute insulin response, which is the first phase of insulin secretion. We also observed that following pancreatectomy, insulin release reached a plateau. Nevertheless, there was no change in insulin secretion throughout the oral glucose challenge. As previously described in this species,^61^ the loss of pancreatic mass would have been balanced by an increase in glucose and GLP‐1 driven insulin secretion per islet. Although we did not measure it in our study, subtotal pancreatectomy may have increased the incretin impact to balance the loss of islet mass.

Contrary to what we expected, the plan to combine a 2‐month HFHSD with a subtotal pancreatectomy in order to exceed the pancreas's capacity for insulin secretion did not result in any phenotypic change. As seen in human islets,^62^ the weight gain brought on by the diet may have helped to increase the size of the surviving islets and their reactivity to glucose in releasing insulin, serving as a mode of compensation.

We found the biggest metabolic changes in the minipigs receiving continuous intraportal glucose and lipid infusions. Even while the findings of changes in insulin response were significantly different from the baseline state only in the group with a subtotal pancreatectomy prior to infusions, both groups—with or without subtotal pancreatectomy—presented comparable patterns. Therefore, we propose that the pancreatectomy potentialized the impact of infusions. In conjunction with a decline in the first phase of insulin secretion, we discovered an increase in hepatic insulin resistance and postprandial hyperinsulinism. Because glucose and lipids were infused into the portal vein, they may have quickly caused a hepatic excess in glycogen and triglycerides, which may have been the source of the hepatic insulin resistance as previously observed in dogs^63^, ^64^ and mice.^65^ Furthermore, the administration of parenteral nutrition is known to have major side effects like hepatic steatosis, insulin resistance, and changes in insulin secretion,^43^, ^66^, ^67^, ^68^ which is why we decided to test this approach in our research. During the sacrifice of these minipigs, a discoloration evocating a hepatic steatosis was macroscopically observed (data not shown).

Additionally, it is now well understood that a decrease in acute insulin response, a marker of change in the first phase of insulin release, constitutes the initial indicator of impaired glucose tolerance.^69^, ^70^ The existence of ectopic triglycerides in the pancreas that were brought on by the intraportal infusions could potentially account for this decline. It is known that ectopic triglycerides have a significant role in the oxidative stress and inflammation that reduce the functionality of pancreatic beta cells.^71^ Around the abdominal organs during sacrifice, substantial visceral adipose tissue was also macroscopically visible (data not shown). This finding, a potential cause of insulin resistance, might thus be used to explain the postprandial hyperinsulinism. Hepatic insulin resistance was clearly established, while peripheral insulin resistance was not. In particular, the HOMA‐IR and Matsuda Index calculations, which evaluate peripheral insulin sensitivity and resistance in humans, did not change after intraportal infusions relative to the initial state (data not shown). In addition, we did not examine postprandial incretin levels. It would have been interesting to determine whether the observed postprandial hyperinsulinism may be attributed to an increase in GLP‐1 concentrations caused by an intestinal adaptation brought on by the intraportal infusions. In any case, the observed modifications would look very similar to those early intervening in the beginning of type 2 diabetes, even if no fasting hyperglycemia or postprandial glucose intolerance were found for these groups. We might have acquired a more severe phenotype if we had continued intraportal infusions for a longer period of time. We did not, however, because of the ethical issues raised by the complicated porcine model.

The lipidic profile of Göttingen‐like minipigs was investigated. All groups showed a notable rise in total cholesterol, especially LDL, with the exception of pigs subjected to a single subtotal pancreatectomy. As a result, we were able to develop a minipig model of the metabolic syndrome in the groups receiving continuous intraportal infusions of glucose and lipids. Although the definition of the metabolic syndrome in pigs is still debatable, the key features of this syndrome in humans include visceral obesity, fasting blood glucose levels over 110 mg/dL, insulin resistance, dyscholesterolemia, hypertriglyceridemia, and elevated blood pressure. Metabolic syndrome is defined as the presence of at least three of these criteria,^72^ which in our instance were at least visceral obesity, insulin resistance, and dyscholesterolemia. Type 2 diabetes and metabolic syndrome were frequently confused in many other studies that worked on developing type 2 diabetic pig models. Because of this, some researchers falsely claimed to have a legitimate preclinical minipig model of type 2 diabetes, despite the fact that the World Health Organization strictly defines diabetes as hyperglycemia and not by a variety of signs of insulin resistance. Minipigs demonstrated both hyperglycemia caused by the toxic medication's use and obesity with metabolic abnormalities in other studies when HFHSD and streptozotocin were combined.^73^, ^74^ However, because metabolic disorders and hyperglycemia are in reality interrelated in the disease's genesis, it was in this case two different independent interventions that produced two phenotypic characteristics independently, raising question on the reliability of this type 2 diabetes paradigm.

Finally, it is intriguing to note that the only intervention that significantly impacted the way that glucose is metabolized was one in which we mimicked an intestinal over absorption of glucose and lipids. Previous research suggested that one of the causes of the onset of type 2 diabetes would be an increase in the intestinal glucose absorption rate.^75^, ^76^ Reciprocally, a study identified several intestinal sodium‐glucose transporter 1 (SGLT1) variants that would be protective against type 2 diabetes and the progression of the metabolic syndrome.^77^ It would be fascinating to see in future research if these SGLT1 variants are largely present in the pig. Additionally, the associated gene might provide a good target for developing genetically altered pig models and researching the effects on glucose metabolism.

Our study presents some limitations. We mentioned in a previous paragraph the probably too short duration of the intraportal glucose and lipids infusion to induce a more severe phenotype. The type‐II error, associated to statistical analyses, could also have prevented us to highlight differences between strains or interventions, although the estimated minimal number of animals in each group was sufficient to demonstrate an effect.

In summary, we were successful in developing a preclinical minipig model with early signs of glucose intolerance and metabolic syndrome, but we were unsuccessful in obtaining a model of type 2 diabetes. Furthermore, the metabolic changes were in line with what had been reported about the disease's early pathogenesis. Thus, the pig continues to be a useful preclinical large animal model for imitating the metabolic syndrome, including insulin resistance, visceral obesity, and dyslipidemia, as we have verified in this work. The minipig, however, has more to contribute as a healthy model, supporting the necessity to choose the proper species for each type of study. The pig's continued difficulty in achieving a fasting hyperglycemia may prompt us to rethink using it as a translational diabetic subject in accordance with the WHO definition of diabetes mellitus.

## AUTHOR CONTRIBUTIONS

**Rébecca Goutchtat:** Conceptualization (equal); data curation (equal); formal analysis (lead); investigation (equal); methodology (equal); project administration (equal); visualization (lead); writing – original draft (lead). **Audrey Quenon:** Data curation (equal); investigation (equal); methodology (equal); project administration (equal). **Manon Clarisse:** Data curation (equal); formal analysis (supporting); investigation (supporting); project administration (supporting). **Nathalie Delalleau:** Investigation (supporting). **Anaïs Coddeville:** Investigation (supporting). **Mathilde Gobert:** Investigation (supporting). **Valéry Gmyr:** Resources (equal); validation (lead); writing – review and editing (equal). **Julie Kerr‐Conte:** Conceptualization (supporting); resources (equal); writing – review and editing (supporting). **François Pattou:** Conceptualization (equal); funding acquisition (lead); resources (equal); writing – review and editing (supporting). **Thomas Hubert:** Conceptualization (lead); funding acquisition (equal); investigation (equal); methodology (lead); project administration (supporting); resources (lead); supervision (lead); writing – review and editing (lead).

## FUNDING INFORMATION

The authors warmly thank the European Genomic Institute for Diabetes (EGID) for the funding and its precious contribution for animal disposal and housing, the Institut National de la Santé et de la Recherche Médicale (Inserm) for the stipend “Poste d'Accueil” and the Agence Nationale de Recherche (ANR).

## CONFLICT OF INTEREST STATEMENT

The authors declare no conflict of interest.

## ETHICAL APPROVAL

Animals were used in this investigation, which received approval from the local French Committee of Animal Research and Ethics (CEEA‐75, n°#18,915) in accordance with European law (2010/63/EU directive) and the widely accepted ARRIVE guidelines. The agreed‐upon (n°D59‐35010) Département Hospitalo‐Universitaire de Recherche et d'Enseignement (Dhure) in Lille, France, was where all the procedures were carried out.

## Data Availability

The data that support the findings of this study are available from the corresponding author upon reasonable request.
